# POPULATION PREVALENCE OF MOTOR FUNCTION IMPAIRMENTS IN US OLDER ADULTS: THE NATIONAL HEALTH AND AGING TRENDS STUDY

**DOI:** 10.1093/geroni/igae098.1195

**Published:** 2024-12-31

**Authors:** Anis Davoudi, Hang Wang, Ryan Dougherty, Amal Wanigatunga, Alden Gross, Jennifer Schrack

**Affiliations:** Johns Hopkins University, North Bethesda, Maryland, United States; Johns Hopkins University, Baltimore, Maryland, United States; Rutgers University, Stoughton, Wisconsin, United States; Johns Hopkins Bloomberg School of Public Health, Baltimore, Maryland, United States; Johns Hopkins Bloomberg School of Public Health, Baltimore, Maryland, United States; Johns Hopkins Bloomberg School of Public Health, Baltimore, Maryland, United States

## Abstract

Motor function impairments reflect physical health status for older adults, which has profound implications for quality of life. However, there is a significant research gap concerning comprehensive, nationally representative data on the prevalence of these impairments, including diminished gait speed, chair stand ability, standing balance, and grip strength, especially among those aged □80 years. We used data from Round 12 of the National Health and Aging Trends Study (NHATS), a nationally representative panel study of U.S. Medicare beneficiaries aged ≥65 years who completed objective physical function testing. Motor function was assessed using gait speed, chair stand speed, standing balance, and grip strength and impairment(s) were defined using standard clinical thresholds or previously published norms. Age was categorized in 5-year intervals [65-69, 70-74, 75-79, 80-84, 85-89, and 90+]. In this US-representative sample of 5055 older adults (mean age: 78.7 years old (78.3-79.1), 54.4% women), weighted prevalence of impairments in gait speed, chair stand speed, standing balance, and grip strength were higher with advancing age. Among those aged 65-69 years, chair stand impairment was the most prevalent (42.4% (37.0%-47.8%)), while gait speed impairment was the most prevalent among those ≥90 years (90.4% (86.0%-94.9%)). Prevalence of being impaired in all four motor function domains was 11.8% (8.5%-15.2%) at ages 65-69 years and 64.0% (58.0%-69.9%) among those ≥90 years. These findings indicate a higher prevalence of impairments in motor function with older age, underscoring the importance of addressing these impairments earlier in life to enhance the quality of life in the aging population.

